# Two decades of post-graduate training in Applied Public Health: The experience and challenges of the Uganda Public Health School Without Walls

**Published:** 2011-12-15

**Authors:** Raymond Tweheyo, Christine Nalwadda, Nicholas Ayebazibwe, David Mukanga, Elizeus Rutebemberwa, William Bazeyo

**Affiliations:** 1 Makerere University School of Public Health, College of Health Sciences (MaKSPH), Uganda; 2 African Field Epidemiology Network (AFENET), Kampala, Uganda

**Keywords:** FETP, postgraduate, training, public health, field epidemiology, Uganda

## Abstract

The objective of this work is to describe the experience of the Uganda Public Health School Without Walls (PHSWOW) in training public health professionals at post-graduate level to offer leadership in planning, delivery of health services and research within a decentralized health system. As one of the constituents of the Makerere University College of Health Sciences, the Uganda PHSWOW has the vision of becoming a Centre of Excellence, providing leadership in public health and the mission of promoting the attainment of better health of the people in Uganda and beyond through public health training, research and community service. Key to the successes of the program are the 238 program graduates, most of whom have remained in-country to serve at district and national levels of service delivery. Collaborations have been established with government, private, non-governmental and international institutions leading to increased health service provision and research for the improvement of health status of populations and influence on public policy. There is still a lot to do in diversifying the skills mix of graduates and contributing to an ambitious increment from 0.4 to 4.7 public health professionals per 10,000 population; as is currently the case in high-middle income countries. Currently, the Uganda PHSWOW has exceeded the proposed output for FETPs of training 3 to 5 graduates per 1 million population suggested by some authors, however the output is still inadequate. More also needs to be done to promote a culture of publication in an effort to translate public health evidence into policy and practice.

## Introduction

The evidence indicating that public health professionals are essential for leading health systems and programs into curtailing the global causes of poor health is extensive [1]. It is well known that in order to tackle the burden of ill health at population level, public health professionals are required to strengthen health systems and manage the bottle necks [1,2]. Research and practice efforts have defined the role of the public health profession as that of a multi-disciplinary team of personnel with technical skills and responsibility for leading health systems at all levels to improve health through a population focus [1–3]. In the mid 1980's Roemer argued that it was necessary to increase schools of public health globally from 100 to 450; each to serve, at least, a population of 10 million [4]. At the time, majority of the schools existed as departments or branches within schools or faculties of medicine, pharmacy, dentistry, or veterinary with the majority established in the United States of America [1,4]. To date, there is an increased number of public health training institutions in low income countries, although in Africa, there is still limited capacity with just over 53 training institutions at postgraduate level, over half with less than 10-member full time faculty, and a total enrolment of under 100 post-graduate students [2,5].

The health status of the population in Uganda has improved over the past two decades between 1991 to 2010 as measured by two indicators; under-five mortality rate from 203 to 137 per 1,000 live births and maternal mortality ratio from 527 to 435 per100,000 live births [6–9]. However, during the same time period, the population has nearly doubled from 16.7 million to an estimated 30.6 million people [7]. The population growth may negate the gains in health service delivery and prompts an urgent need to strengthen the public health workforce to operate health systems in addition to provision of essential medicines, supplies and infrastructure [10]. In recognition of the critical need for increasing the number of public health professionals in developing countries, the Rockefeller Foundation in partnership with the School of Public Health and Tropical Medicine at Tulane University initiated the Public Health Schools Without Walls (PHSWOW) program with input from faculty at Harvard and Johns Hopkins Schools of Public Health [11,12]. The PHSWOW concept aimed to design a 2-year Master of Public Health Program to increase capacity in developing countries for training postgraduate level public health personnel that can apply technical, managerial, and leadership competences to run decentralized health systems.

PHSWOW modeled MPH programs in Africa were initiated in Zimbabwe in 1993, Uganda in 1994 and Ghana in 1995. Public health programs have been since developed with interest in service-provision or practice. A typology of field based public health training programs is described among others by Weis et al and TEPHINET [13,14] as; Epidemic Intelligence Service (EIS) of the Centers for Disease Control and Prevention in USA, the Field Epidemiology Training Programs (FETP) that became apparent in Africa, Asia and South-America after 2000 and the European Programme for Intervention Epidemiology Training (EPIET). Elsewhere, PHSWOW and FETPs are termed Applied Epidemiology Training and Service Programs (AETPs) [15].

To date the Ugandan MPH program modeled on PHSWOW has been successful in achieving its primary objectives. For instance, many of the graduates remain to manage public and private health services [16] with over 85% presence in the about 120 districts country-wide [16]. However, there are new needs for health planning and management of health systems including the use of evidence based public health practice and research. There is a growing need for public health practitioners in the public, private, civil society and international sectors.

Where should current public health training focus? Beaglehole and Dal Poz [17] question whether governments and therefore training institutions should focus on increasing the numbers of the public health workforce, or whether they should in fact focus on broadening the capacity of the public health workforce including; the size, composition, skills mix to enhance performance in dealing with priority health problems. This article contributes to the body of knowledge from the experience of a major post-graduate level public health training institution in Africa.

### Organization of the Uganda PHWOW

Makerere University School of Public Health (MaKSPH) is one of the four constituents of Makerere University College of Health Sciences (MakCHS) created in 2007, others are; School of Health Sciences, School of Medicine and School of Biomedical Sciences. MaKSPH is headed by a Dean and has five departments namely; Department of Health Policy, Planning and Management (HPPM), Department of Epidemiology and Biostatistics, Department of Community Health and Behavioral Sciences, Department of Disease Control and Environmental Health and Regional Centre for Quality of Health Care. The PHSWOW is hosted by the HPPM department similar to other post-graduate programs which are run by a designated department. The program is headed by a director and coordinated with the support of other personnel; the academic coordinator, field coordinator, resident mentor and program administrator. Training of post-graduate students draws expertise from the 40 man staff establishment and additional research fellows and honorary lecturers. The School has a vision of becoming a “Centre of excellence, providing leadership in Public Health”. With the mission; “To promote the attainment of better health for the people of Uganda and beyond through Public Health Training, Research and Community service, with the guiding principles of Quality, Relevance, Responsiveness, Equity and Social Justice” [18]

### Program description and scope

About 60% of the PHSWOW training is field based; where students are assigned to districts that provide practicum training through field mentors, supervisors and faculty supervisory visits. These sites host students as part of the district health team and they are mentored to participate and lead district health efforts and research during their stay. The other 40% of the 2-year Masters duration is didactic with courses covering the whole breadth of the public health discipline. It includes the five core public health trainings of epidemiology, biostatistics, environmental health, health services administration/ management and social and behavioral sciences as described by the US National Academy of Sciences [1]. In addition, other content areas that are covered include; research methods, health informatics, communication, health policy and financing and public health ethics. With the beginning of 2010/2011, trainees have the option to elect additional specialty courses in applied epidemiology, health systems, environmental health or reproductive health. In the earlier years of the program, training for students was donor-funded from notably, Rockefeller Foundation, WHO, Italian Cooperation including tuition, field placement and scholastic materials, however, in line with development partnership agreements, funding reduced in 2004. The CDC has supported the field work, epidemic investigations and field coordination since 2002.

### Program intake

As of 2011 Uganda program has had 17 cohorts with a total of 238 graduates. In the earlier years following program inception, the annual enrollment averaged 10 up to 1999. The enrollment rose to 18 in 2000, 21 in 2001 and to 36, its highest in 2002. Since then, numbers have ranged between 15 and 24 (average 20), with the target capacity set at 30. There are currently 39 trainees in the program, 21 in year 1 (2011/2012 intake) and 18 in year 2 (2010/2011 intake).

## Outputs-addressing service needs

### Training outputs

The 238 graduates of the program occupy a broad range of positions in the public health landscape both nationally and internationally. In Uganda these range from senior ministry of health management positions, public health program managers, district health managers, NGOs and, private health facilities. Internationally the program's graduates are spread across Africa, Asia, the United Kingdom and Oceania.

### Research outputs

Throughout the 2-year program, trainees are tasked to develop and conduct two short field studies and a dissertation. The field studies should address priority health problems or managerial gaps within the district health system and are vetted by the District Health Officer [19]. The dissertation on the other hand is an academic requirement that undergoes rigorous scientific scrutiny to conform to set standards; scientific merit and contribution to addressing an existing public health knowledge or practice gap. At least two supervisors are allocated per trainee who may later support the trainee into publication of findings. In the past 10 years, trainees have increasingly written manuscripts from their dissertations supported by their supervisors, for example, in the areas of epidemiology [20], health systems [21], maternal health [22,23] and more recently increased involvement with faculty in general research and publication [24]. Publications from students are still limited and this is an output that has not been thoroughly exhausted since there is no standard or benchmark. On the side of faculty, Nankinga [25] quantified an increase in publications within the Makerere University College of Health Sciences, where the PHSWOW is a key contributor from 100 in 2005 to double the number in 2009. This research potential can be harnessed and trainees mentored through the MPH training to gain the discipline and skills for research dissemination to scientific and policy making fora. With support from AFENET and other partners to MaKSPH, at least one third of the trainees are involved in dissemination of research findings at regional and international fora. For instance in 2010 five trainees presented at the 14th International Congress on Infectious Diseases in Florida, Miami, three presented at the 59^th^ annual meeting of the American Society of Tropical Medicine and Hygiene in Atlanta, and an additional nine trainees presented at the 6th Global TEPHINET Scientific Conference in Cape Town, South Africa [26,27]. These scientific abstracts present an opportunity for further development into publishable manuscripts if resources could be devoted to this cause. Graduates of the program have also been engaged in several ground breaking research projects locally and internationally including HIV/AIDS vaccine trials, Highly Active Anti-Retroviral Therapy efficacy studies, medical male circumcision studies, childhood illness research and demographic health surveys [26,27].

### Service provision outputs

MaKSPH collaborates with Ministry of Health (MoH), District Health Offices, District local governments, International and non-governmental organizations in planning and implementation of health programs. MaKSPH has a total of 15 district field training sites where students work with district health teams to identify health problems and address them through evidence based approaches. Students participate in surveillance activities and investigation of disease outbreaks in the districts and the region as a whole including Ebola, cholera, Marburg heamorrhagic fever, meningococcal meningitis, Hepatitis E and nodding disease. In 2010, every trainee was involved in at least one such field activity. Several trainees and graduates were actively involved in the 2001 Ebola epidemic response in Uganda [28]. For example, one of the program graduates, Dr. Mathew Lukwiya, notified the nation and the whole world of this deadly disease [28]. He however, contracted the viral hemorrhagic fever while attending to the patients and later succumbed to the disease. The MaKSPH under MaKSPH-CDC HIV Fellowship Program recognizes the best fellow every year with a Lukwiya Award in his honour.

## Collaborations

The Program has developed collaborative relationships with several multilateral agencies including the World Bank, European Union, the Division for International Development (DFID) and UN agencies like WHO, UNHCR, WFP and Gates Foundation, and National Institute of Health (NIH) As a result of these collaborations, the MaKSPH has a long-standing experience in conducting community and population-based studies in designated study sites/labs including; the Rakai Health Sciences Program (RHSP), the Kayunga Cohort Development (CODE) study of the Makerere University Walter Reed Project (MUWRP), and the Iganga-Mayuge Health Demographic Surveillance Site (HDSS) [26,27].

The Uganda program is a founding member of the African Field Epidemiology Network (AFENET)a networking alliance of all FETPs and PHSWOWs in Africa. The program hosted AFENET in its first year of existence. The Uganda program continues to be an active member of the network and works closely with AFENET in responding to acute public health needs in Uganda and the region at large. With support from AFENET, the MaKSPH and Institute of Tropical Medicine, Antwerp graduates in Uganda formed an alumni association in March 2011. The association is meant to facilitate further linkage between the training institution and the research and practice outputs of alumni. Inadequacy of the linkage between training, research and community service has been identified [2]. MaKSPH has got numerous long-standing capacity building and research collaborations with Universities such as Karolinska Institutet, London School of Hygiene and Tropical Medicine, Harvard School of Public Health, Johns Hopkins School of Public Health, Tulane University, Case Western Reserve University, University of Alberta, Swiss Tropical Institute, Muhimbili University of Health and Allied Sciences, School of Public Health, National University of Rwanda (NUR), University of Western Cape South Africa, Jimma University Ethiopia, Kinshasa University School of Public Health, School of Public Health Moi University, University of Bergen, Norway and other reputable institutions around the world. The collaborations are a means to enhance capacity and effectiveness in research and community service giving opportunities for capacity development to faculty and program staff within projects.

## Future challenges for attention

One key challenge that exists for the program is to address the skills mix of public health graduates. Already, in 2010/2011 academic year, MaKSPH has embarked on a revised curriculum where trainees elect a track in which additional courses are offered; applied epidemiology, health systems, environmental or reproductive health. This is expected to increase the skills mix to match the relevant needs of the health system, however its implementation and outputs deserve attention through process evaluations to assess their effectiveness.

The critical mass for public health training is still far from being achieved considering research and practice needs. As opposed to the current 0.4 public health professionals per 10,000 population in Uganda, there is need to target achieving 4.7 public health professionals per 10,000 population similar to the situation in high-middle income countries [29]. Some authors have suggested that FETPs should train at least 3 to 5 graduates per million inhabitants [30,31] translating into 170 graduates from the Ugandan program. This target has been exceeded without realizing the critical numbers of practitioners. There is need for further research on adequacy and skill mix of public health workforce in low income settings. Tracking alumni and increasing research outputs of trainees and graduates is another major challenge. Since research in community sites/labs focuses on pertinent policy challenges within health system, students that select an elective should have their dissertation benefit from a natural home within such an existing institutional research lab. On the other hand, student dissertations are currently not accepted to be conducted on secondary or historical data, yet such data is extremely relevant for evaluation of health interventions. If these conditions are waived, an upsurge in student and faculty research outputs in the form of publications is likely.

The greatest challenge to the training program is that of strengthening field placement in the era of limited partner funding. There are challenges pertaining to student choice of and maintenance of field placement sites due to infrastructural and system development resource needs. We suggest that institutional research sites should be optimally utilized as student field placement labs. We also suggest that hat the Uganda PHSWOW needs to seek external funding through grants to maintain the quality and number of field sites to ensure that the PHSWOW training continues to address the health priorities of the Ugandan public.

**Table 1:** Number of trainees involved in partner-led field activities, June 2010 – July 2011

Activity	Date	Number of trainees involved in Investigation/ Response	Lead Agency and support partner
Martyr's Day Celebration Medical Surveillance	June 2011	05	AFENET
Yellow Fever Vaccination Coverage Survey, 5 districts in Northern Uganda	May/June 2011	07	WHO
Viral Haemorrhagic Fever Surveillance	March 2011 to date	02	CDC
One Health Central and East (OHCEA) Monitoring and Evaluation, Baseline Survey	July 2011	03	USAID
National Yellow Fever Epidemic Reactive Vaccination (5 districts)	January/ February 2011	05	MoH
Cholera Outbreak Investigation	March 2011	05	AFENET
Anthrax Response Review Meeting	March 2011	05	AFENET
Participatory Epidemiology Investigation of under-five diarrhea in Kisumu, Kenya	October, November 2010	03	ILRI, CDC
Risk Assessment for Anthrax among Game-Reserve Communities	September 2010	02	AFENET/ USAID
Global Health Institute (International Course focusing on ONE HEALTH)	August 2010	03	University of Minnesota
Participatory Epidemiological investigation on Nodding disease	August 2010	02	AFENET/ CDC

ILRI: International Livestock Research Institute; MoH: Ministry of Health, Uganda. Source: Quarterly reports of the MPH PHSWOW to AFENET Secretariat, 2010 – 2011
